# Assessment of knowledge of primary healthcare physicians in the western region of Saudi Arabia about Hidradenitis Suppurativa

**DOI:** 10.3389/fmed.2025.1483112

**Published:** 2025-05-09

**Authors:** Jumana Nabeel Akbar, Mawaddah Abdulgader Tallab, Sarah Bandar Aljoudi, Houriah Y. Nukaly, Mohammed Haitham Abduljabbar

**Affiliations:** ^1^Faculty of Medicine, King Abdulaziz University, Jeddah, Saudi Arabia; ^2^Department of Dermatology, King Fahad General Hospital, Jeddah, Saudi Arabia; ^3^Department of Dermatology, King Abdulaziz University Hospital, King Abdulaziz University, Jeddah, Saudi Arabia; ^4^Faculty of Medicine, Batterjee Medical College, Jeddah, Saudi Arabia

**Keywords:** acne inversa, general physicians, physician training, clinical competence, primary healthcare, skin lesions

## Abstract

**Background:**

Hidradenitis Suppurativa (HS) is a relatively common chronic inflammatory skin disorder. Early identification by primary healthcare (PHC) physicians plays a crucial role in preventing its adverse outcomes.

**Objective:**

To assess the knowledge of primary healthcare physicians in the Western Region of Saudi Arabia regarding HS and identify the determinants influencing their knowledge level.

**Methods:**

A cross-sectional study was conducted among randomly selected PHC physicians working in the cities of Jeddah, Makkah, and Taif. A valid and reliable self-administered online questionnaire was used for data collection.

**Results:**

A total of 106 PHC physicians participated in the study, with ages ranging from 26 to 48 years. The majority (79.2%) were aware of HS. The most frequently reported source of information was a diagnosed patient (64.3%). Overall, 47.2% of participants demonstrated a good level of knowledge regarding HS. Female physicians (*p* = 0.043), those over 35 years of age (*p* = 0.001), family medicine consultants (p < 0.001), physicians with more than 10 years of clinical experience (*p* = 0.008), and those who had seen more than five dermatological cases per day (*p* = 0.019) were significantly more knowledgeable. Most participants assigned the responsibility for diagnosis, treatment, and follow-up of HS cases to dermatologists, followed by surgeons or PHC physicians. About one-third (33%) had previously diagnosed a case of HS.

**Conclusion:**

Although most PHC physicians in Western Saudi Arabia were aware of HS, less than half demonstrated a good level of knowledge, and only one-third had ever diagnosed a case. These findings highlight the need to implement targeted dermatology education programs for PHC physicians.

## 1 Introduction

Hidradenitis Suppurativa (HS) is a chronic inflammatory skin disorder with a prevalence rate ranging from < 1% to 4%. Its clinical course is variable, ranging from mild to severe cases (1). It is characterized by its occurrence frequently, and mostly affecting axillary, inguinal, and perianal regions (2), and can affect multiple sites at the same time. The disease is characterized by painful subcutaneous nodules end up by fibrosis and sinuses affecting the apocrine gland (3). The disease is almost double affecting the females than males and usually undiagnosed in early stages. Early identification of HS by primary healthcare physicians has a significant role in preventing its adverse effects (4). The pathophysiology of the disease is not well known till now, however many theories tried to explain it, including a theory of blockage of the apocrine opening which result in an expansion of the glands and create a suitable environment for bacterial infection followed by microbial infection and gland burst then the infection cover all the subcutaneous tissues (1). Some limited studies have been conducted in Saudi Arabia and outside it showed insufficient knowledge about HS diagnosis and management among primary care physicians, particularly general practitioners in Jeddah city (4–6). The aim of the study is to assess the knowledge of primary healthcare physicians in Western Region-Saudi Arabia regarding HS and identify the determinants of their knowledge level.

## 2 Material and methods

### 2.1 Study design and participants

This study adopted a cross-sectional design and was conducted online, targeting primary healthcare (PHC) physicians working in the Western Region of Saudi Arabia, specifically in the cities of Jeddah, Makkah, and Taif. The study population included all PHC physicians employed at primary healthcare centers and hospitals in these cities. Eligibility criteria required participants to have worked for at least 1 year post-graduation, with no restrictions on nationality, and to not be involved in administrative roles. There were no specific exclusion criteria. A sample size of 143 physicians was determined using the online Raosoft sample size calculator, based on a 5% margin of error, a 90% confidence level, and a 50% response distribution. A convenience non-probability online sampling technique was used to select the sample.

### 2.2 Measures

Data were collected through a valid and reliable self-administered questionnaire, for which permission was obtained from the original author. The questionnaire consisted of three main sections. The first section gathered sociodemographic data, including age, gender, nationality, job title, years of clinical experience, previous experience in dermatology clinics, and the average number of dermatological cases seen per day. The second section assessed the physicians' general knowledge of Hidradenitis Suppurativa (HS). Participants were provided with high-resolution images of HS and asked to identify the disease. Participants were shown a total of four high-resolution clinical images, each representing a different dermatological condition. Only one image correctly depicted Hidradenitis Suppurativa, while the remaining three represented other common skin diseases. This approach was designed to assess the participant's ability to distinguish HS from other dermatoses in a realistic clinical context. They were also asked if they had heard of HS before, their source of information, whether they had ever diagnosed HS, and if so, the number of cases seen. The final section included six questions with yes, no, or “I don't know” responses regarding the features of HS, followed by five questions testing the physicians' beliefs about which specialty should manage and follow up with HS patients (dermatology, family medicine, general surgery, plastic surgery, or “I don't know”) and their overall confidence in diagnosing and treating HS.

### 2.3 Statistical analysis

Data were entered and analyzed using the Statistical Package for Social Sciences (SPSS) software, version 26. The knowledge assessment included six statements and one picture, with correct answers scored as “1” and incorrect or “don't know” answers scored as “0.” The total score and its percentage were calculated, with participants scoring 60% or above classified as having “good knowledge” and those scoring below 60% classified as having “poor knowledge.” Descriptive statistics, including frequency and percentage for categorical variables, and mean and standard deviation for continuous variables, were used to summarize the data. The association between HS knowledge level and other categorical variables was tested using the Chi-square test, with a *p*-value of < 0.05 considered statistically significant.

### 2.4 Ethics statement

This study was conducted in accordance with the Declaration of Helsinki and the ethical approval for the study was obtained from the Regional Research and Ethics Committee in Makkah. Additionally, approval was sought from the directors of primary healthcare in Jeddah, Makkah, and Taif before the distribution of the questionnaire to the participants. Before answering the questionnaire, we included the following informed consent statement: “You are invited to participate in a research study on Hidradenitis Suppurativa (HS). This research aims to gain a better perspective on the knowledge and of primary healthcare physicians regarding HS among primary healthcare physicians in the Western Region of Saudi Arabia. Participation is voluntary and anonymous. If you agree to participate in this study, you can start answering the questionnaire.”

## 3 Results

One hundred and six primary healthcare physicians were included in the study out of targeted 143, giving a response rate of 74.1%. Table 1 summarizes their demographic and work-related characteristics. Their age ranged between 26 and 48 years with a mean ± SD of 31 ± 4.7 years. Females represent 55.7% of them. Majority were Saudi nationals (99.1%). More than half of them (53.7%) were family medicine residents whereas 12.3% were family medicine consultants. Almost two-thirds of them (68.9%) has a practical clinical experience of 5 years or less after graduation. Most of them (70.8%) reported previous rotation in dermatology clinics; among almost half of them (48%) this was since more than 1 year. More than one-third of them (36.8%) have seen on average 1–3 dermatological cases/day.

**Table 1:** Demographic and work-related characteristics of the participants (*n* = 106).

**Variable**	**Frequency**	**Percentage**
**Gender**		
Male	47	44.3
Female	59	55.7
**Age in years**		
≤ 30	68	64.2
31–35	21	19.8
>35	17	16.0
**Range**	26–48	
**Mean** **±standard deviation**	31.0 ± 4.7	
**Nationality**		
Saudi	105	99.1
Non-Saudi	1	0.9
**Job title**		
General practitioner	15	14.2
Family medicine resident	57	53.7
Family medicine specialist	21	19.8
Family medicine consultant	13	12.3
**Years of clinical practice**		
≤ 5	73	68.9
6–10	25	23.6
>10	8	7.5
**Previous rotation in dermatology clinic**		
Yes	75	70.8
No	31	29.2
**Duration of rotation in dermatology clinics (*****n** =* **75)**		
≤ 1 month	34	45.3
2 months	20	26.7
≥3 months	21	28.0
**Last time of rotation in dermatology clinics (*****n** =* **75)**		
This year	18	24.0
Last year	21	28.0
>One year	36	48.0
**Average number of dermatology cases/day**		
< 1	9	8.5
1–3	39	36.8
4–5	27	25.5
>5	31	29.2

### 3.1 Awareness about Hidradenitis Suppurativa

**Figure 1:**
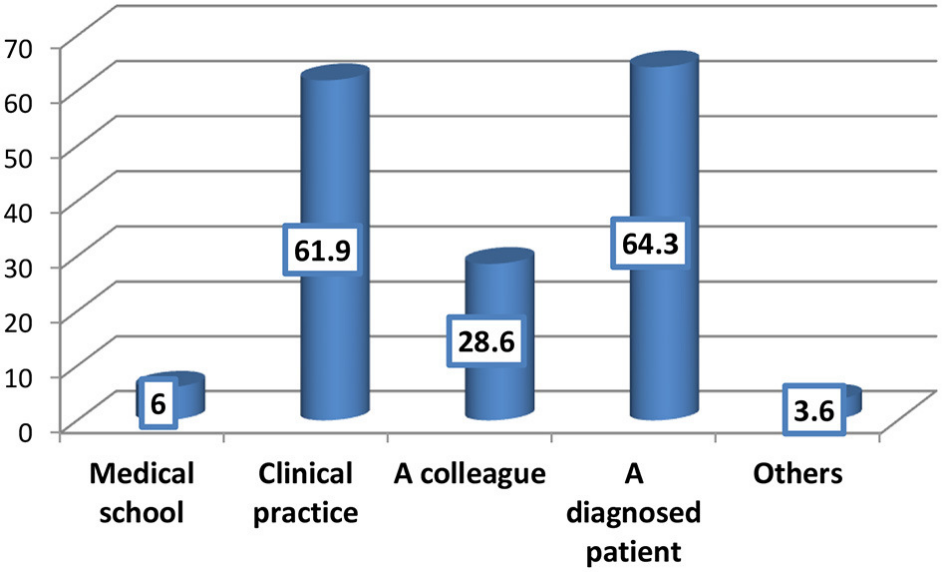
Source of awareness about Hidradenitis Suppurativa among the participants (*n* = 84).

**Figure 2:**
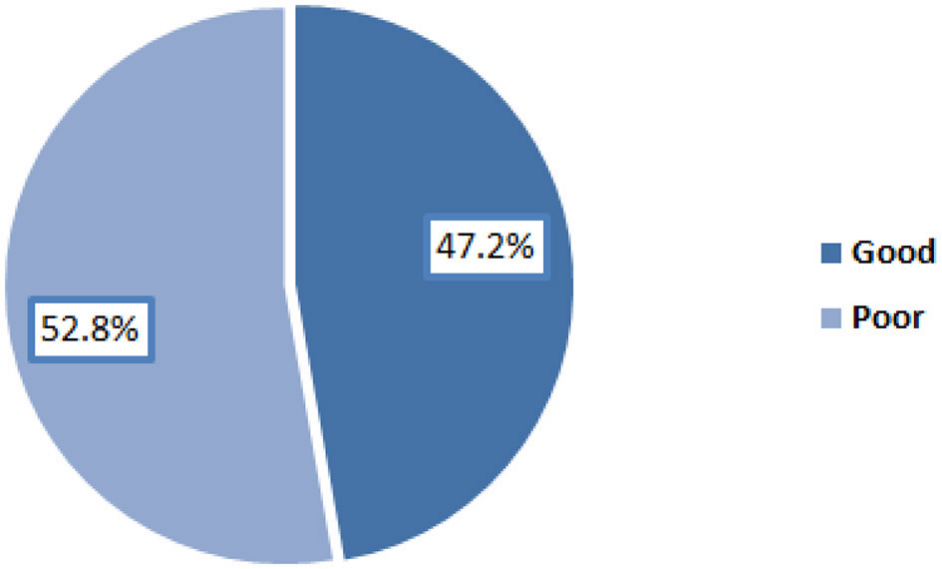
Level of knowledge about Hidradenitis Suppurativa among the participants.

Most of the primary healthcare physicians (79.2%) were aware of HS as shown in Figure 1; among them, the most frequent reported sources of information about HS were a diagnosed patient (64.3%) and clinical practice (61.9%) (Figure 2).

### 3.2 Knowledge about Hidradenitis Suppurativa

Almost half of the PHC physicians (50.9%) could recognize the picture describing a case of Hidradenitis Suppurativa, out of 4 pictures. Regarding HS clinical manifestations, most of them (79.2%) knew correctly that HS occurs with lesions typically localized in the following regions: axillary, inter-inframammary, inguinal, perineal, gluteus. Almost two-thirds knew that HS manifests with abscesses (67%), draining fistulas (65.1%) and inflammatory nodules (60.4%). Less than half of the PHC physicians could recognize that HS manifests with scars (45.3%) (Table 2).

**Table 2:** Participants' responses to knowledge pictures and statements about Hidradenitis Suppurativa.

**Knowledge statements and questions**	**Correct answer**		
	**Answer**	**Number**	**%**
Which picture best describe Hidradenitis Suppurativa?	C	54	50.9
HS manifest with painful skin lesions	Yes	62	58.5
HS manifest with inflammatory nodules	Yes	64	60.4
HS manifests with abscesses	Yes	71	67.0
HS manifests with draining fistulas	Yes	69	65.1
HS manifests with scars	Yes	48	45.3
HS occurs with lesions typically localized in the following regions: axillary, inter-inframammary, inguinal, perineal, gluteus	Yes	84	79.2

Overall, 47.2% of the participants had good level of knowledge regarding Hidradenitis Suppurativa as illustrated in Figure 2.

Female physicians had higher rate of good level of knowledge about HS compared to male physicians (55.9% vs. 36.2%), *p* = 0.043. Physicians aged over 35 years expressed the highest rate of good knowledge about HS (88.2%) whereas those aged 30 years or less had the lowest rate (36.8%), *p* = 0.001. All family medicine consultants compared to only 6.7% of general practitioners had good level of HS knowledge, *p* < 0.001. For the benefit of international readers, it is important to clarify that in Saudi Arabia, family medicine consultants are senior physicians who have completed advanced specialty training in family medicine and are board-certified, often holding supervisory or leadership roles. In contrast, general practitioners (GPs) typically hold a basic medical degree without specialization and have not undergone structured residency training in family medicine. This difference in training and clinical exposure likely explains the significant knowledge gap observed in our study. All physicians with clinical experience of more than 10 years compared to only 42.5% of those with experience of 5 years or less had good level of HS knowledge, *p* = 0.008. About two-thirds of physicians who have seen >5 dermatological cases (61.3%) compared to only 11.1% of those who have seen < 1 patient/day expressed good level of HS knowledge, *p* = 0.019 (Table 3).

**Table 3:** Factors associated with primary healthcare physicians' knowledge about Hidradenitis Suppurativa.

**Variable**	**Level of knowledge about Hidradenitis Suppurativa**		*p*-value^*^
	**Poor**	**Good**	
	***N** =* **56**	***N** =* **50**	
	***N*** **(%)**	***N*** **(%)**	
**Gender**		
Male (*n =* 47)	30 (63.8)	17 (36.2)	
Female (*n =* 59)	26 (44.1)	33 (55.9)	0.043^*^
**Age in years**		
≤ 30 (*n =* 68)	43 (63.2)	25 (36.8)	
31–35 (*n =* 21)	11 (52.4)	10 (47.6)	
>35 (*n =* 17)	2 (11.8)	15 (88.2)	0.001^*^
**Nationality**		
Saudi (*n =* 105)	56 (53.3)	49 (46.7)	
Non-Saudi (*n =* 1)	0 (0.0)	1 (100)	0.472^**^
**Job title**		
General practitioner (*n =* 15)	14 (83.3)	1 (6.7)	
Family medicine resident (*n =* 57)	34 (59.6)	23 (40.4)	
Family medicine specialist (*n =* 21)	8 (38.1)	13 (61.9)	
Family medicine consultant (*n =* 13)	0 (0.0)	13 (100)	< 0.001^*^
**Years of clinical practice**		
≤ 5 (*n =* 73)	42 (57.5)	31 (42.5)	
6-10 (*n =* 25)	14 (56.0)	11 (44.0)	
>10 (*n =* 8)	0 (0.0)	8 (100)	0.008^*^
**Previous rotation in dermatology clinic**		
Yes (*n =* 75)	38 (50.7)	37 (49.3)	
No (*n =* 31)	18 (58.1)	13 (41.9)	0.488^*^
**Duration of rotation in dermatology clinics (*****n** =* **75)**			
≤ 1 month (*n =* 34)	17 (50.0)	17 (50.0)	
2 months (*n =* 20)	12 (60.0)	8 (40.0)	
≥3 months (*n =* 21)	9 (42.9)	12 (57.1)	0.545^*^
**Last time of rotation in dermatology clinics (*****n** =* **75)**			
This year (*n =* 18)	5 (27.8)	13 (72.2)	
Last year (*n =* 21)	12 (57.1)	9 (42.9)	
>One year (*n =* 36)	21 (58.3)	15 (41.7)	0.083^*^
**Average number of dermatology cases/day**			
< 1 (*n =* 9)	8 (88.9)	1 (11.1)	
1-3 (*n =* 39)	18 (46.2)	21 (53.8)	
4-5 (*n =* 27)	18 (66.7)	9 (33.3)	
>5 (*n =* 31)	12 (38.7)	19 (61.3)	0.019

^*^Chi-square test, ^**^Fischer Exact test.

### 3.3 Thoughts of the participants regarding diagnosis and treatment of Hidradenitis Suppurativa

The responses of the participants regarding their thoughts concerning who is responsible for diagnosis, treatment and follow up of HS cases are summarized in Table 4. Majority of them put responsibility on dermatologists, followed by surgeon or PHC physician.

**Table 4:** Participants thoughts regarding the involved specialty for diagnosis, therapy and follow-up of cases of Hidradenitis Suppurativa.

**Parameter**	**Dermatologist, *N* (%)**	**Surgeon, *N* (%)**	**PHC physicians, *N* (%)**	**Plastic surgeon, *N* (%)**	**Don't know, *N* (%)**
Hidradenitis Suppurativa diagnostic suspicion is supported by:	88 (83.0)	24 (22.6)	30 (28.3)	20 (18.9)	3 (2.8)
he diagnosis of Hidradenitis Suppurativa under which specialty?	88 (83.0)	27 (25.5)	15 (14.2)	19 (17.9)	3 (2.8)
In the Hidradenitis Suppurativa therapy setting, the reference figure^*^ is:	87 (82.1)	36 (34.0)	17 (16.0)	30 (28.3)	4 (3.8)
In the management of drug therapy (topical/systemic), the reference figure is	88 (83.0)	16 (15.1)	13 (12.3)	20 (18.9)	5 (4.7)
In the follow up of Hidradenitis Suppurativa patients, the reference figure is:	90 (84.9)	31 (30.1)	31 (30.1)	26 (24.5)	5 (4.7)

^*^Chi-square test. ^**^Fischer Exact test.

### 3.4 Hidradenitis Suppurativa-related practice

About one-third (33%) of the PHC physicians ever diagnosed a case of HS; among them 34.3% diagnosed more than three cases. Almost two-thirds of them (67.9%) preferred to manage early stage of HS while only 12.3% preferred to treat resistant case of HS. Minority of the participants expressed excellent level of overall confidence in diagnosis (1.9%) and treatment (2.8%) of HS (Table 5).

**Table 5:** Practice of the primary healthcare physicians regarding Hidradenitis Suppurativa.

**Parameter**	**Frequency**	**Percentage**
**History of ever diagnosis of a case of Hidradenitis Suppurativa**		
No	71	67.0
Yes	35	33.0
**Number of diagnosed cases (*****n** =* **35)**		
One	4	11.4
Two	10	28.6
Three	9	25.7
More than three	12	34.3
**Do you prefer to manage early stage of HS?**		
No	11	10.4
Yes	72	67.9
Not sure	23	21.7
**Do you prefer to treat resistant case of HS?**		
No	64	60.3
Yes	13	12.3
Not sure	29	27.4
**Level of o overall confidence in HS diagnosis**		
Excellent	2	1.9
Very Good	22	20.8
Average	56	52.8
Poor	26	24.5
**Level of o overall confidence in HS treatment**		
Excellent	3	2.8
Very Good	11	10.4
Average	61	57.6
Poor	31	29.2

More than half (58.8%) of PHC physicians aged >35 years compared to 26.5% of those aged below 30 years reported history of ever diagnose a case of HS, *p* = 0.040. About two-thirds (69.2%) of family medicine consultants compared to none of general practitioners ever diagnosed a case of HS, *p* = 0.001. Physicians who have seen an average of >5 dermatology cases/day were more likely to report ever diagnosing a case of HS compared to those who have seen < 1 dermatology cases/day (58.1% vs. 22.2%), *p* = 0.006. There was a statistically significant association between knowledge level about HS and history of ever diagnosing a case of the disease among the PHC physicians, *p* < 0.001 (Table 6).

**Table 6:** Factors associated with history of ever diagnosis of a case of Hidradenitis Suppurativa among the participants.

**Variable**	**Ever diagnose a case of Hidradenitis Suppurativa**		*p*-value^*^
	**No**	**Yes**	
	***N** =* **71**	***N** =* **35**	
	***N*** **(%)**	***N*** **(%)**	
**Gender**			
Male (*n =* 47)	33 (70.2)	14 (29.8)	
Female (*n =* 59)	38 (64.4)	21 (35.6)	0.528^*^
**Age in years**			
≤ 30 (*n =* 68)	50 (73.5)	18 (26.5)	
31-35 (*n =* 21)	14 (66.7)	7 (33.3)	
>35 (*n =* 17)	7 (41.2)	10 (58.8)	0.040^*^
**Nationality**			
Saudi (*n =* 105)	71 (67.6)	34 (32.4)	
Non-Saudi (*n =* 1)	0 (0.0)	1 (100)	0.330^**^
**Job title**			
General practitioner (*n =* 15)	15 (100)	0 (0.0)	
Family medicine resident (*n =* 57)	40 (70.2)	17 (29.8)	
Family medicine specialist (*n =* 21)	12 (57.1)	9 (42.9)	
Family medicine consultant (*n =* 13)	4 (30.8)	9 (69.2)	0.001^*^
**Years of clinical practice**			
≤ 5 (*n =* 73)	50 (68.5)	23 (31.5)	
6-10 (*n =* 25)	18 (72.0)	7 (28.0)	
>10 (*n =* 8)	3 (37.5)	5 (62.5)	0.173^*^
**Previous rotation in dermatology clinic**			
Yes (*n =* 75)	47 (62.7)	28 (37.3)	
No (*n =* 31)	24 (77.4)	7 (22.6)	0.142^*^
**Duration of rotation in dermatology clinics (*****n** =* **75)**			
≤ 1 month (*n =* 34)	22 (64.7)	12 (35.3)	
2 months (*n =* 20)	14 (70.0)	6 (30.0)	
≥3 months (*n =* 21)	11 (52.4)	10 (47.6)	0.480^*^
**Last time of rotation in dermatology clinics (*****n** =* **75)**			
This year (*n =* 18)	14 (77.8)	4 (22.2)	
Last year (*n =* 21)	15 (71.4)	6 (28.6)	
>One year (*n =* 36)	18 (50.0)	18 (50.0)	0.086^*^
**Average number of dermatology cases/day**			
< 1 (*n =* 9)	7 (77.8)	2 (22.2)	
1-3 (*n =* 39)	30 (76.9)	9 (23.1)	
4-5 (*n =* 27)	21 (77.8)	6 (22.1)	
>5 (*n =* 31)	13 (41.9)	18 (58.1)	0.006^*^
**Knowledge about Hidradenitis Suppurativa**			
Poor (*n =* 56)	50 (89.3)	6 (10.7)	
Good (*n =* 50)	21 (42.0)	29 (58.0)	< 0.001^*^

^*^Chi-square test, ^**^Fischer Exact test.

## 4 Discussion

Hidradenitis Suppurativa is a disease characterized by a very long delay in diagnosis, reaching up to several years which lead to impairment of the patient's clinical status and quality of life (7).

Primary healthcare physicians are in front line facing these patients. Therefore, their role in early diagnosis and management of the disease is essential to prevent the adverse outcomes (8–10). In this context, the present study was done to assess primary healthcare physicians' awareness, and knowledge regarding HS diagnosis and management in Western Saudi Arabia.

Majority of the PHC physicians in the present study (79.2%) were aware of HS, which is an encouraging finding putting in mind the rarity of the disease in Saudi Arabia with very limited studies were carried out concerning the disease (2, 11). The main source of information about HS in the present survey was a diagnosed patient (64.3%), followed by clinical practice (61.9%). In another similar Saudi study carried out in Jeddah, q little bit different results were identified as 71.2% of participants have heard about HS, and their main sources of information were clinical practice (39.4%), followed by medical school (21.2%) (5).

About half of the PHC physicians expressed good level of knowledge about HS in the present study. The same has been observed in a recent study carried out in Jeddah (KSA) (5). Better level of knowledge has been observed in a study carried out in Denmark and Belgium among general practitioners (GPs) (12). However, in a similar recent study conducted among primary healthcare physicians in Portugal, overall insufficient knowledge regarding HS was observed (6), despite the fact that HS is more common in Europe than in our region.

In the present study, female physicians were more knowledgeable about HS compared to male physicians. However, gender difference was not observed in another Saudi study (5). Older physicians (>35 years) were more knowledgeable about HS than younger physicians in the present study. In another similar Saudi study (5), physicians' age was not significantly associated with HS knowledge level. As expected, family medicine consultants and those with more years of practice expressed higher rate of good knowledge about HS compared to their peers. Alhawsawi et al. (5) reported a significant association between current job title and medical degree of primary healthcare physicians and their knowledge level about HS. On the other hand, having rotation in dermatology department was not associated with knowledge about HS. This finding put question mark on the quality of training given to PHC physicians during the dermatological rotation. This observation may reflect a broader gap in the undergraduate and postgraduate medical education system in Saudi Arabia, where dermatology is often underrepresented in the curriculum. Dermatology rotations, when available, are usually short and lack hands-on exposure to chronic and complex conditions like HS. This limited exposure may prevent physicians from developing confidence in diagnosis and management, despite formal training.

With increasing the number of dermatology cases seen, the level of good knowledge about HS increased among PHC physicians in the current study. In a similar study carried out in Jeddah among primary healthcare physicians, knowledge level about HS was significantly associated with history of ever previously diagnosis of the disease (5). Comparison between studies, although very few and using the same tool, is not practical due to variation in the demographics of the participants.

In the present study, majority of the PHC physicians put responsibility for diagnosis, treatment and follow up of HS cases on dermatologists, followed by surgeons and PHC physicians. The same has been reported by others (5).

In addition to its dermatological manifestations, Hidradenitis Suppurativa is increasingly recognized as a systemic disease with significant comorbidities. HS has been associated with metabolic syndrome, obesity, insulin resistance, cardiovascular disease, and various psychiatric conditions such as depression and anxiety. These associations underscore the need for a multidisciplinary approach in managing HS, involving dermatologists, endocrinologists, cardiologists, and mental health professionals. Raising awareness of these systemic implications among PHC physicians is essential for comprehensive patient care and early intervention strategies that go beyond the skin (10, 13, 14).

The results highlight an urgent need to strengthen dermatology education at both undergraduate and postgraduate levels in Saudi Arabia. Integrating case-based discussions, interactive modules, and clinical exposure to dermatological conditions like HS could improve physician preparedness. Additionally, offering continuing medical education (CME) workshops focused on common and underdiagnosed skin disorders may bridge current knowledge gaps among PHC physicians. These steps would empower PHC physicians to play a more active role in early diagnosis and management, potentially reducing referral burden and improving patient outcomes (14).

Limitations of the present study include the fact that very few studies were identified locally as well as internationally, which limit the ability to compare findings of this study with others. Also, carrying out this study among PHC physicians in Western Saudi Arabia could impact the generalizability of findings over other regions of the Kingdom. Despite of those limitations, up to our knowledge, this study explored topic rarely and included an acceptable sample size with good response rate.

## 5 Conclusion

Although most of the primary health care physicians in Western Saudi Arabia were aware of Hidradenitis Suppurativa, less than half of them expressed good level of knowledge about the diseases; particularly females, older (>35 years), more experienced, consultants and those have seen a greater number of dermatological cases per day. Furthermore, about one-third of PHC physicians ever diagnosed a case of HS, particularly older (>35 years), consultants and those have seen a greater number of dermatological cases per day. There was an association between the level of knowledge about HS and its diagnosis among the PHC physicians. Majority of the PHC physicians put responsibility on dermatologists, followed by surgeons and PHC physicians regarding HS management. Based on the study's findings, we recommended encouragement of PHC physicians to take their responsibility in diagnosis and management of HS, and organizing educational activities with dermatology consultant to increase awareness and knowledge of PHC physicians regarding various dermatological disorders, including HS. Future publications and presentations should emphasize ongoing developments in research methodologies and healthcare practices. Highlighting such progress not only reflects the dynamic nature of medical research but also reinforces the importance of translating these insights into improved patient care outcomes, particularly in Jeddah and comparable healthcare settings.

## Data Availability

The datasets presented in this study can be found in online repositories. The names of the repository/repositories and accession number(s) can be found in the article/Supplementary material.
