# Supplementary material for: Assessment of knowledge of primary healthcare physicians in the western region of Saudi Arabia about Hidradenitis Suppurativa
Source: Front Med (Lausanne). 2025 May 9;12:1483112. doi: 10.3389/fmed.2025.1483112 (PMC12098085; doi:10.3389/fmed.2025.1483112)
Supplement: Supplementary file 1 [file Table_1.docx]

**Assessment of Knowledge of Primary Healthcare Physicians in Western Region Saudi Arabia About Hidradenitis Suppurativa**

| **1. What is your age?**   1. )Personal Data : | …………… years |
| --- | --- |
| **2.Gender:** | 1. Male 2. Female |
| **3.Nationality:** | 1. Saudi 2. Others(specify):…………….. |
| **4. Job title:** | 1. General practitioner.   2. Family medicine resident  3. Family medicine specialist  4. Family medicine consultant  4. Others |
| **5. Years of clinical practice** | ……………...years |
| **6. Any previous experience in derma clinic :**  **6.a )duration :**  **6.b ) last time :** | 1. yes ,if yes go to 6.a and 6.b  2. No , if no go to 7  ………………...  1. This year .  2. Last year .  3. > 1 year |
| **7. Number of Dermatology Cases Seen per Day** | 1. < 1 2. 1 - 3 3. 4 - 5 4. > 5 |
| 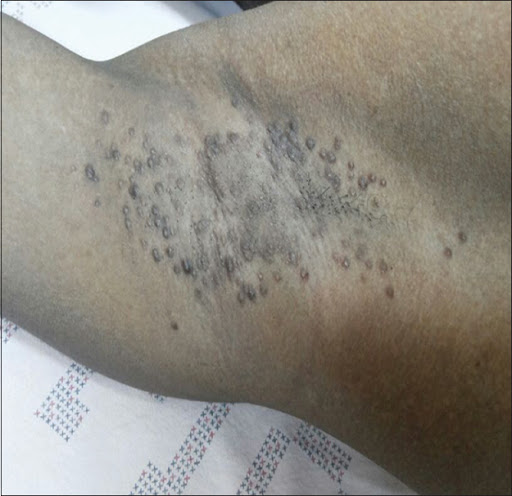  B  A  **A. Look at the picture ,**  **Which of them is best describehidradenitis suppurativa?**  **B.** | 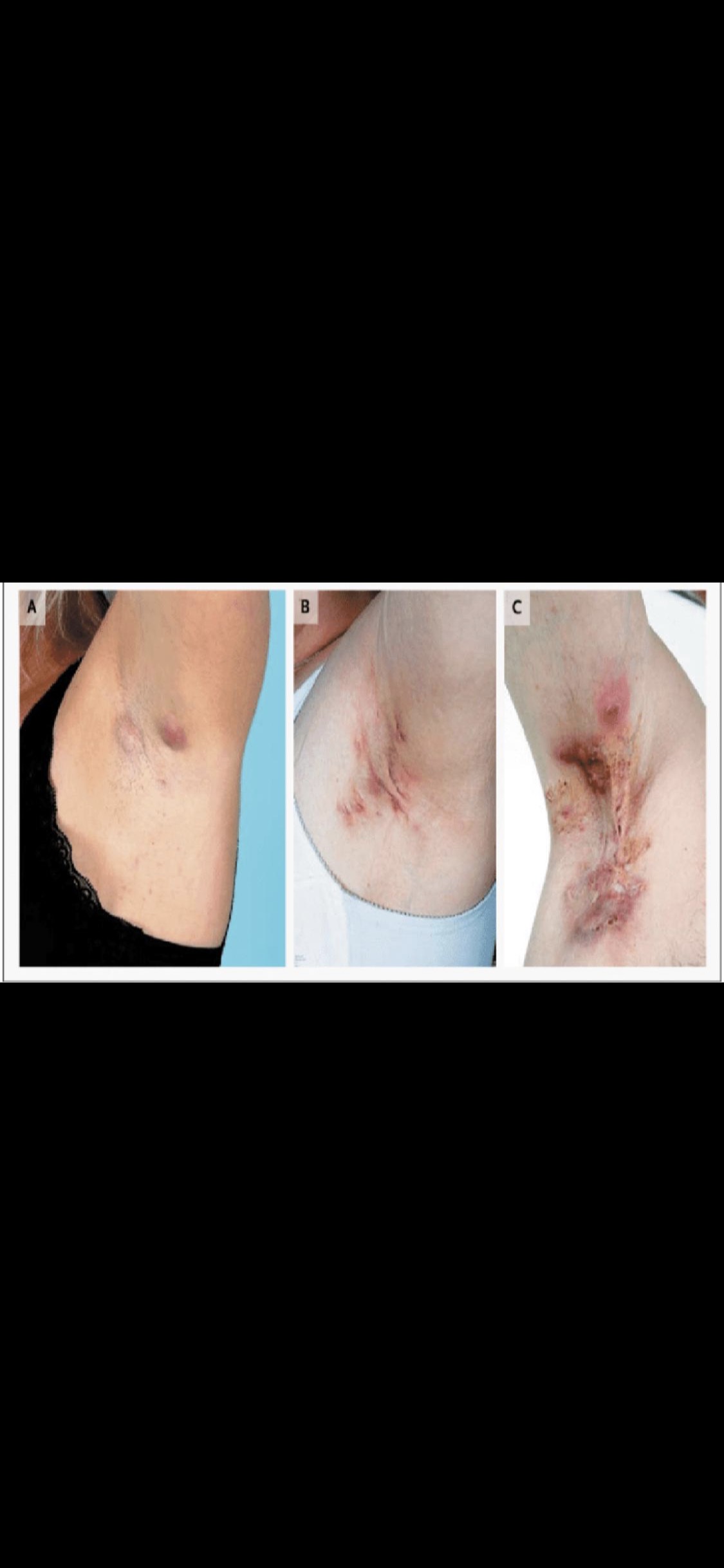  C   1. A 2. B 3. C 4. D |

| 1. Do you know about hidradenitis suppurativa (HS)? | 1. Yes 2. No   *(if NO skip the next question)* |
| --- | --- |
| 2. From where you get your knowledge about hidradenitis suppurativa  (you can choose more than one answer) | 1. Medicalschool 2. Clinicalpractice 3. Acolleague 4. A diagnosedpatient 5. Others(specify):………………………. |
| 3. Have you ever diagnosed any case with hidradenitis suppurativa? | 1. Yes 2. No   *(if NO skip the next question )* |
| 4. How many cases? | …………………….. |


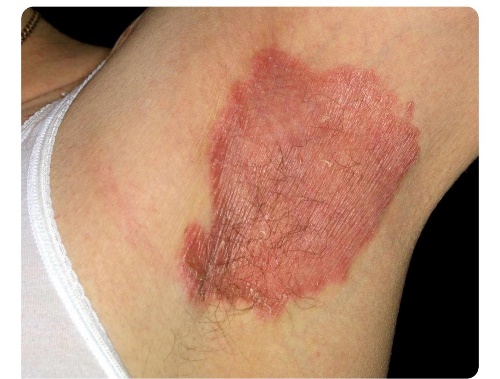

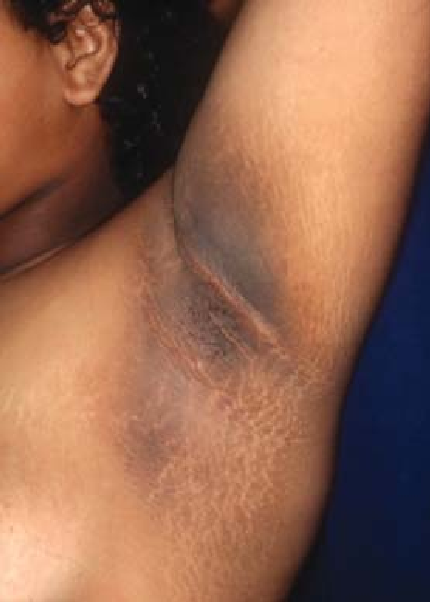


D

1. ) knowledge:

**c.**

| *select one best answer* | Yes | No | I don’t  know |
| --- | --- | --- | --- |
| 1. HS manifest with painful skin lesions |  |  |  |
| 2. HS manifest with inflammatory nodules |  |  |  |
| 3. HS manifests with abscesses |  |  |  |
| 4. HS manifests with draining fistulas |  |  |  |
| 5. HS manifests with scars |  |  |  |
| 6. HS occurs with lesions typically localized in the following regions: axillary, inter-inframammary, inguinal, perineal, gluteus |  |  |  |

| *you can choose more than one answer* | Dermatologist | Surgeon | Primary healthcare  physician | Plastic surgeon | I Do Not know |
| --- | --- | --- | --- | --- | --- |
| 1. hidradenitis suppurativa diagnostic  suspicion is supported by: |  |  |  |  |  |
| 2. The diagnosis of hidradenitis suppurativa  under which specialty? |  |  |  |  |  |
| 3. In the hidradenitis suppurativa therapy  setting, the reference figure* is: |  |  |  |  |  |
| 4. In the management of drug therapy (topical/systemic), the reference figure is |  |  |  |  |  |
| 5. In the follow up of hidradenitis suppurativa  patients, the reference figure is: |  |  |  |  |  |

1. ) Practice :

reference figure ( responsible physician )

Do you prefer to manage early stage of HS?

1. Yes .

2. No.

3. Not Sure.

Do you prefer to treat resistant case of HS?

1. Yes .

2. No.

3. Not Sure.

Are you over all confident in HS diagnosis? 1. Excellent .
 2. Very Good .
 3. Average .

4. Poor .

Are you over all confident in HS treatment? 1. Excellent .
 2. Very Good .

3. Average .

4. Poor .
